# HU protein is involved in intracellular growth and full virulence of *Francisella tularensis*

**DOI:** 10.1080/21505594.2018.1441588

**Published:** 2018-02-23

**Authors:** Pavla Stojkova, Petra Spidlova, Juraj Lenco, Helena Rehulkova, Lucie Kratka, Jiri Stulik

**Affiliations:** Department of Molecular Pathology and Biology, Faculty of Military Health Sciences, University of Defence, Hradec Kralove, Czech Republic

**Keywords:** DNA binding protein, *Francisella*, FPI, virulence, HU protein, nucleoid-associated protein

## Abstract

The nucleoid-associated HU proteins are small abundant DNA-binding proteins in bacterial cell which play an important role in the initiation of DNA replication, cell division, SOS response, control of gene expression and recombination. HU proteins bind to double stranded DNA non-specifically, but they exhibit high affinity to abnormal DNA structures as four-way junctions, gaps or nicks, which are generated during DNA damage. In many pathogens HU proteins regulate expression of genes involved in metabolism and virulence. Here, we show that the *Francisella tularensis* subsp. *holarctica* gene locus FTS_0886 codes for functional HU protein which is essential for full *Francisella* virulence and its resistance to oxidative stress. Further, our results demonstrate that the recombinant FtHU protein binds to double stranded DNA and protects it against free hydroxyl radicals generated via Fenton's reaction. Eventually, using an iTRAQ approach we identified proteins levels of which are affected by the deletion of *hupB*, among them for example *Francisella* pathogenicity island (FPI) proteins. The pleiotropic role of HU protein classifies it as a potential target for the development of therapeutics against tularemia.

## Introduction

*Francisella tularensis* (*F. tularensis*), the pathogenic intracellular bacterium, is causative agent of zoonotic disease tularemia. Its ability to resist in a phagocytic cell through phagosomal escape and replication in the cytosol is the main aspect of its virulence [1]. On molecular level several irreplaceable factors have been identified: specific cell wall structure consisting of lipopolysaccharide [2–4], capsule [5], and virulence proteins which are encoded on the FPI [6]. However, many other proteins, not only those from the FPI, are required for *F. tularensis* virulence. As we show below, one of them is nucleoid associated protein often called HU protein, which was predicted as a novel candidate for virulence factor previously [7].

In general, HU proteins belong to histone like proteins (share some properties with eukaryotic histones) [8], sometimes called nucleoid associated proteins (for their localization in nucleosome) [9]. In *E. coli*, and other enterobacteria, HU protein is present mainly as a heterodimer consisting of two subunits, HU-α and HU-β, encoded by *hupA* and *hupB* [8]. In other bacteria such as *Mycobacterium tuberculosis* HU protein is homodimer, consisting of only one subunit – HU-β, encoded by *hupB* [10]. Similarly, only *hupB* was found in *Francisella* genome.

HU proteins often manifest high affinity to damaged DNA which occurs when bacteria have to overcome host defense tools in order to prosper itself. Consequently, production of the virulence factors encoded by virulence genes is triggered. The effect of HU protein on the expression of virulence genes has been reported in *Salmonella enterica* serovar Typhimurium [11] and *Porphyromonas gingivalis* [12]. HU protein in *E. coli* plays an important role in the initiation of DNA replication [13], cell division, SOS response [14,15] and galactose metabolism [16]. It controls 8 % of the whole *E. coli* genome. These genes are associated with adaptation to the unfriendly host environment or with a stress response [14]. Noteworthy, in the context of *M. tuberculosis*, this protein is potential target for the development of therapies against tuberculosis [10].

*Wolbachia* HU protein could be also involved in regulation of host environment, as proposed by Beckmann *et al*. These authors suggest that acidic/basic motifs at the N terminus are recognized by secretion system type IV (T4SS) and thus HU can be accumulated in host nucleus and thereby could bind to mosquito sperm DNA. This idea is supported by structural and functional similarity between bacterial HU and eukaryotic HMG proteins [17]. HU protein is secreted outside bacterial cell, as demonstrated in *Pseudomonas* study, where the HU protein is component of extracellular matrix and is part of biofilm [18]. Histone like proteins could bind to host DNA, but they also could play a role in adhesion of bacterial cell through collagen binding in epithelial respiratory cells [19]. In the context of *F. tularensis*, HU protein was found to be secreted to the culture filtrate [20].

These interesting findings inspired us to investigate HU protein as a potential *Francisella* virulence factor. Indeed, in our study we showed participation of HU protein in virulence of *F. tularensis* both, *in vitro* and *in vivo*. We proved its role in DNA protection against free hydroxyl radicals, in resisting the oxidative stress and using proteomic approach we identified number of proteins affected by *hupB* deletion, of which the most interesting are the FPI proteins.

## Results

### HU is necessary for efficient intramacrophage replication

**Figure 1.**
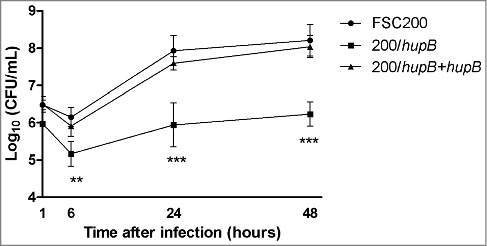
HU protein is a prerequisite for effective *Francisella* proliferation inside BMMs. Significant difference in ability of intracellular growth is evident at 6 h after infection. Mutant strain is not able to replicate in murine macrophages as effectively as both WT and complemented strains. P value < 0.05 *, *P* < 0.01 **, *P* < 0.001 ***, *P* < 0.0001 ****.

Ability to survive and replicate within hostile intracellular milieu of phagocytic cells is the main feature of *Francisella* species. In order to verify an importance of *hupB* for *Francisella* intracellular life we used as target cells bone marrow-derived macrophages. These cells were infected with WT strain; mutant and complemented mutant strains at MOI of 50. Infected cells were cultivated for 1 h, 6 h, 24 h and 48 h. In particular time points after infection the cells were lysed and number of intracellular bacteria was calculated by CFU counts. Significant differences between WT and deletion mutant strains were observed (Fig. 1). The first relevant variance was detected already 6 h after infection. However, the most significant differences were observed 24 h and 48 h after infection. The number of viable deletion mutant bacteria increased by 5.9 and by 6.2 log_10_ for 24 h and 48 h time interval, respectively. Whereas the CFU counts for WT increased by 7.9 and 8.2 log_10_. This intra-macrophage growth defect was eliminated by complementation of deletion mutant strain *in trans*. The complemented strain replicated comparably to the WT strain in all tested intervals after infection. This finding demonstrates that the HU protein plays an important role in intracellular multiplication of *Francisella* microbes.

### FSC200/*hupB* mutant is attenuated in *in vivo* model of infection and elicits protective immune response against WT challenge

**Figure 2.**
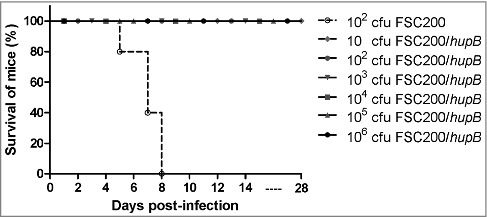
FSC200/*hupB* mutant is attenuated in *in vivo* model of tularemia. Groups BALB/c mice, 6–10 weeks old, were infected with 200 μl of bacterial suspension subcutaneously (s.c.) with appropriate infection doses of 10^1^, 10^2^, 10^3^, 10^4^, 10^5^ and 10^6^ CFU/mouse for FSC200/*hupB* mutant and 10^2^ CFU/mouse for FSC200 and complemented strain FSC200/*hupB+hupB*. Mice infected by FSC200 died within 8 days after infection. In contrast, neither increasing infection doses of FSC200/*hupB* cause the death of mice.

To determine whether the FSC200/*hupB* is also attenuated *in vivo* we compared the survival of BALB/c mice (five mice/group) infected with either mutant or WT strains. Control group was infected subcutaneously with 10^2^ CFU of fully virulent FSC200 strain and the remaining groups with 10, 10^2^, 10^3^, 10^4^, 10^5^ and 10^6^ CFU of FSC200/*hupB* mutant. The mice were observed for morbidity and mortality through a period of 28 days. As was expected all mice infected with WT strain died, on the other hand all mice inoculated with mutant strain remain alive (Fig. 2). Likewise in *in vitro* studies complementation *in trans* led to the restoration of WT phenotype (data not shown).

The potential of FSC200/*hupB* to elicit protective immune response was further tested. The survivors from previous experiment were challenged with WT at a dose of 10^2^ CFU/mouse on day 30 post-infection. All immunized mice survived the WT challenge and displayed no symptoms of tularemia disease during the following 3 weeks. These results revealed that FSC200/*hupB* mutant is able to fully protect BALB/c mice against the virulent FSC200 strain even at the vaccination dose of 10 cfu of mutant bacteria.

### Proteomic analyses

**Figure 3.**
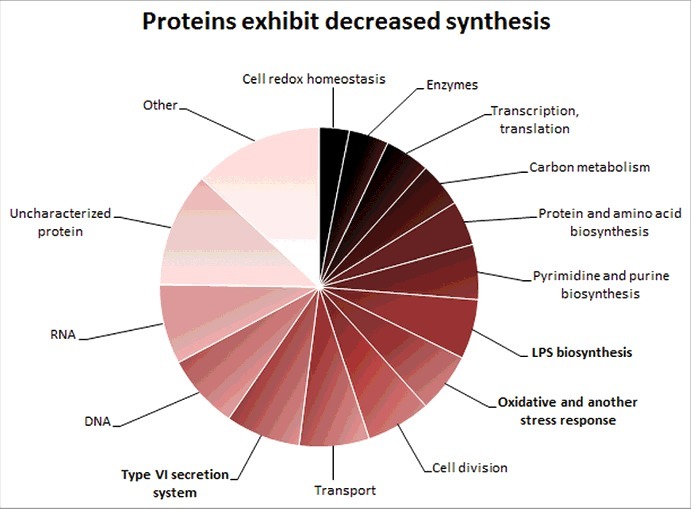
Proteins exhibiting decreased synthesis. Diagram shows groups of proteins with reduced synthesis in *hup*B null mutant that were detected in iTRAQ analysis. The number of these proteins is indicated in the brackets. Associated with: RNA (16), DNA (15), Type VI secretion system (15), transport (14), cell division (13), oxidative and another stress response (12), LPS biosynthesis (12), pyrimidine and purine biosynthesis (11), protein and amino acid biosynthesis (9), carbon metabolism (9), transcription and translation (9), enzymes (8), cell redox homeostasis (6) and other [FeS cluster assembly (6), tryptofan biosynthesis (5), panthothenate biosysnthesis (3), vitamin B (3), glycerol metabolism (3), pilus assembly (3), kinases (2), antibiotic response (1)].

To investigate the molecular mechanism of the mutant attenuated phenotype we compared the proteome of the mutant strain with its WT counterpart using semi-quantitative comparative approach based on iTRAQ peptide labeling. iTRAQ quantitative analysis revealed that deletion of *hupB* had a huge impact on proteomic profile in mutant bacteria. The levels of almost a quarter of proteins were significantly changed (198 proteins with significantly decreased synthesis and 244 proteins with significantly increased synthesis (Table S4)). All FPI proteins but IglG (IglG was not identified at all) were found among proteins that exhibit decreased synthesis (Fig. 3) in *hupB* null mutant strain. When we looked at known FPI regulators, namely MglA, SspA and PigR proteins, iTRAQ analysis showed that there is no significant change related to former two proteins. Concerning PigR the situation was completely different. PigR did not fulfill the criteria set for evaluation (protein quantification with at least two peptides in both replicates) and thus was not taken into account. PigR was quantified in both analyses but each time only with one, distinct peptide.

**Figure 4.**
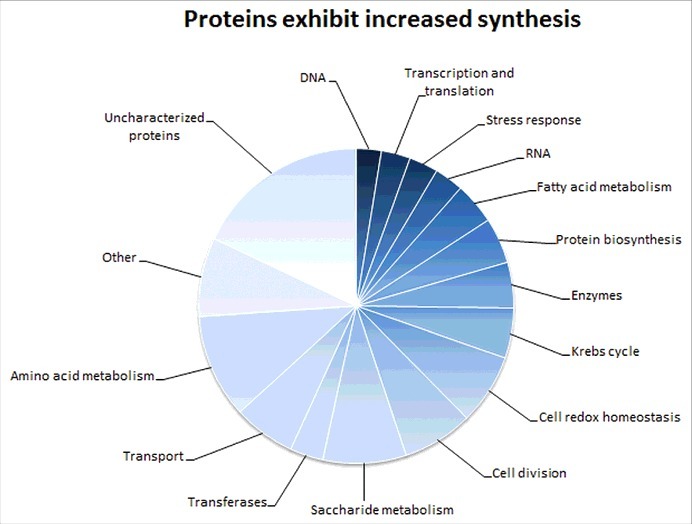
Proteins exhibiting increased synthesis. Diagram shows groups of proteins with increased synthesis in *hup*B null mutant that were detected in iTRAQ analysis. The number of these proteins is indicated in the brackets. Associated with: Amino acid metabolism (25), transport (23), saccharide metabolism (20), cell division (17), cell redox homeostasis (17), Krebs cycle (12), enzymes (11), protein biosynthesis (11), fatty acid metabolism (10), RNA (7), stress response (7), transcription and translation (7), DNA (6), lipid metabolism (3) and other [pyrimidine and purine biosynthesis (5), nucleotide metabolism (7), antibiotic response (1), capsule and LPS (2), cyanophycin (2), terpenoid (2), vitamin biosynthesis (3), iron and nitrogen (4)].

Besides FPI proteins, many other proteins with decreased synthesis implying the important HU role in metabolic processes; such as for example cell redox homeostasis, LPS biosynthesis and stress response, were identified. Among proteins with increased synthesis, for example, RNA binding protein Hfq, that acts as a global regulator of gene expression in stress tolerance and pathogenesis [21] and was found to be a positive regulator of FPI genes [22] could be mentioned. Further e.g. the MoxR protein, a member of a highly conserved class of chaperones that may contribute to *Francisella* pathogenesis and is also implicated in regulation of citric acid cycle [23]; and many others which are involved in carbohydrate metabolism, lipid and amino acid biosynthesis and proteins related to stress response (Fig. 4).

**Figure 5.**
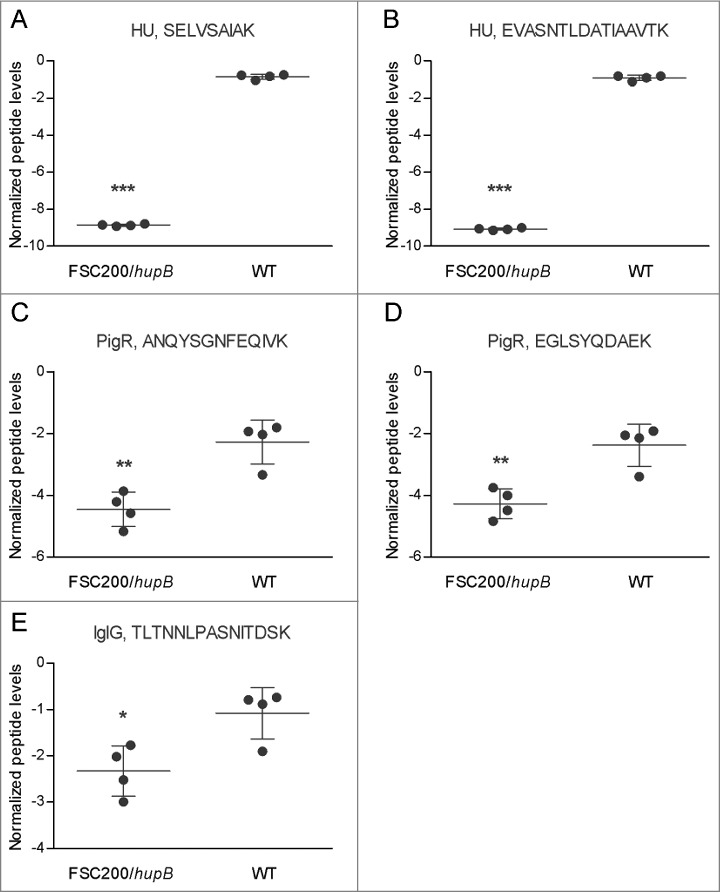
PigR and IglG exhibit decreased synthesis in FSC200/*hupB* mutant. Targeted quantification approach was used for evaluation of PigR and IglG protein levels. Normalized peptide levels for (A) HU peptide SELVSAIAK, (B) HU peptide EVASNTLDATIAAVTK, (C) PigR peptide ANQYSGNFEQIVK, (D) PigR peptide EGLSYQDAEK; and (E) IglG peptide TLTNNLPASNITDSK are shown. PigR exhibits significantly decreased synthesis in the mutant strain in the contrast to WT. P value < 0.05 *, P < 0.01 **, *P* < 0.001 ***, *P* < 0.0001 ****.

To complement results from iTRAQ analysis and to focus on interesting proteins that i) were not identified (IglG) or ii) did not fulfill the set criteria for evaluation (PigR) we performed targeted proteomic analysis. In contrast to exploratory analyses working in a stochastic manner to identify and possibly relatively quantify as many proteins from the complex mixture as possible, target LC-MS assays are intended to specifically detect and accurately and reproducibly quantify only few or several proteins of interest. Targeted quantification analysis proved the significantly decreased synthesis of IglG protein as well as PigR in FSC200/*hupB* mutant strain (Fig. 5). These results thus corroborate the findings from iTRAQ analysis and support the importance of HU protein in FPI expression.

### *pigR* and FPI genes show reduction on transcription level

**Figure 6.**
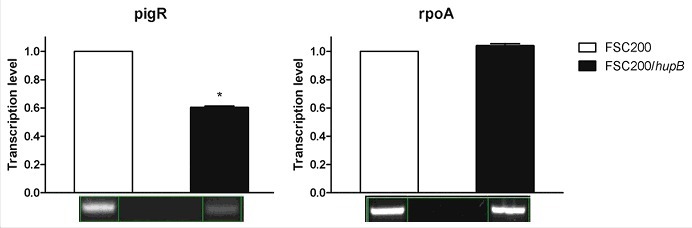
Semi-quantitative RT-PCR demonstrates decreased transcription level of *pigR* gene. Expression of *pigR* was verified on transcription level using reverse transcription followed by PCR. Samples were analyzed by gel electrophoresis and intensities of bands were determined by Bio1D software (CertainTech). *pigR* showed significantly decreased expression in mutant strain in contrast to WT, whereas the transcription level of *rpoA* remained unchanged. This result suggests HU participation in *pigR* regulation. P value < 0.05 *, P < 0.01 **, *P* < 0.001 ***, *P* < 0.0001 ****.

In order to verify the iTRAQ data on transcription level we performed reverse transcription-PCR of *pigR* and FPI genes. Protein level of RpoA was not altered thus the transcription level of *rpoA* was chosen as a control. In line with our expectation the *pigR* (Fig. 6) and almost all of FPI genes (Fig. S1) showed significantly decreased expression in mutant strain in contrast to WT, whereas the transcription level of *rpoA* remained unchanged. Taken together with iTRAQ data these results confirm role of HU protein in *pigR* and FPI genes expression.

### LPS analysis

iTRAQ analysis also revealed the decreased synthesis of 10 proteins in *hupB* null mutant strain which are involved in biosynthesis of O-antigen. Therefore, we decided to verify if the biosynthesis of LPS is affected in this mutant strain. Production of LPS was visualized using Pro-Q Emerald 300 Gel staining (Invitrogen) of hot phenol-water extracted LPS (Fig. S2) and using anti-LPS antibody (Fig. S3). *F. tularensis* subsp. *holarctica* FSC200/*wbtDEF::Cm* lacking LPS was used as a negative control. The typical ladder-like pattern of O-antigen was detected in the FSC200/*hupB* mutant but in comparison to WT strain with lower intensity in the middle part of the ladder. Apparently, at least one of the O-antigen subunits was lost (Fig. S2).

### FSC200/*hup*B mutant is less resistant to human serum than WT

**Figure 7.**
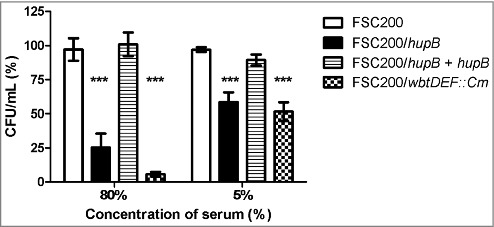
FSC200/*hup*B mutant is less resistant to human serum than WT. FSC200, FSC200/*hupB*, FSC200/*hupB*+*hupB* and FSC200/*wbtDEF::Cm* strains were incubated with human nonimmune serum and their resistance/susceptibility was determined by CFU counts. FSC200/*wbtDEF::Cm* strain is highly susceptible to the effects of complement and the strain was used as a positive control for serum complement activity. Data are presented as percent survival (i.e., CFU obtained from incubations in a serum normalized to CFU from incubations in PBS). Means ± SD from three independent experiments are given. Mutant strain shows less resistance to human serum than WT and complemented strain. P value < 0.05 *, P < 0.01 **, *P* < 0.001 ***, *P* < 0.0001 ****.

Taken into account that the LPS biosynthesis seems to be affected in *hupB* mutant strain and the fact that the alterations in LPS reflect in higher sensitivity to complement-mediated lysis, we tested the ability of mutant strain to resist the human serum. WT, *hupB* mutant strain, the complemented mutant strain, and the FSC200/*wbtDEF::Cm* strain were exposed to 5% and 80% of human nonimmune serum for 90 min at 37°C. The numbers of resistant bacteria were calculated using CFU counts. The significant sensitivity of FSC200/*wbtDEF::Cm* strain to human serum on the one hand and the resistance of FSC200 on the other hand were consistent with previously published data [24,25]. The *hupB* mutant strain also exhibits the high degree of sensitivity to complement-mediated lysis. In the case of 5% human serum 41% of mutant bacteria were killed, when using 80% human serum only 25% of bacteria survive (Fig. 7). Mutant strain complemented *in trans* showed resistance to effects of serum comparable to WT level.

### Oxidative stress conditions are limiting for *hupB* null mutant strain

**Figure 8.**
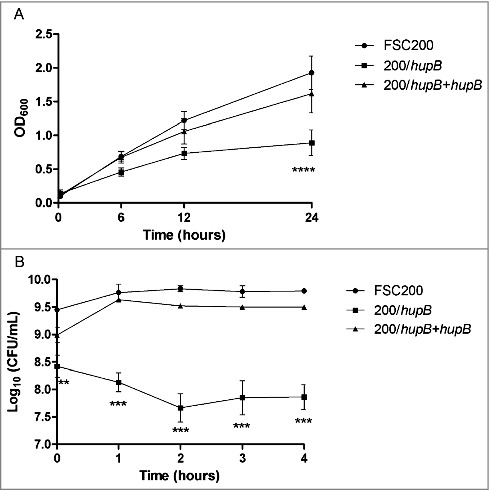
FSC200/*hupB* mutant is more sensitive to oxidative stress stimuli than WT. Oxidative stress survival. (A) Bacteria were grown in the presence of 20 μM CuCl_2_ and the optical density (600 nm) was measured using microplate reader FLUOstar Optima. Significant difference in growth was evident after 24 h measurement. (B) Oxidative stress induced using 0.03% hydrogen peroxide. The bacteria were plated on chocolate agar plates at different times after H_2_O_2_ addition, and viable bacteria were monitored 3 days after. Mutant growth is limited by oxidative stress conditions induced by both CuCl_2_ and H_2_O_2_. P value < 0.05 *, P < 0.01 **, *P* < 0.001 ***, *P* < 0.0001 ****.

Another group of proteins which were found by iTRAQ that exhibit decreased synthesis in the mutant encompassed small proteins and enzymes engaged in cell redox homeostasis. Hence we tested the capability of the mutant strain to resist oxidative stress conditions induced by 20 μM CuCl_2_ or 0.03% hydrogen peroxide. Both assays showed decreased ability of mutant strain to grow in these conditions. First, recording the optical density (600 nm) was used for observation of the mutant ability to grow in complete Chamberlain medium (standard growth conditions) (Fig. S3), and in Chamberlain medium supplemented with 20 μM CuCl_2_ (stress growth conditions) (Fig. 8A). The very significant difference in case of growth in oxidative stress was detected after 24 h measurement, when the mutant strain was not able to grow as effectively as both WT and complemented mutant strains. On the other hand there were no significant differences between mutant and WT strain when grown in standard growth conditions (Fig. S4).

Second, the survival in H_2_O_2_-induced oxidative stress conditions was tested. Bacteria were cultivated in Chamberlain medium to OD_600_ of approx. 1 and then H_2_O_2_ at final concentration of 0.03% was added. In particular time points (1 h, 2 h, 3 h and 4 h) the aliquots of bacterial cultures were diluted and plated on McLeod agar. Viable bacteria were enumerated by CFU counting. Logarithms of obtained values were plotted to the graph (Fig. 8B). It is important to mention that the log values of live bacteria at the time zero (corresponding to OD_600_ = 1) differ between the mutant and WT strains in the range of one order. Lower viability (up to one logarithm) of the mutant strain in Chamberlain medium at the same optical density as WT was confirmed by flow cytometry using LIVE/DEAD® BacLight™ Bacterial Viability and Counting Kit (Thermo Fisher Scientific, data not shown). However, although the mutant and WT do not have the same number of live bacteria at the start of the experiment, logarithmic decline of mutant bacteria is evident.

### FtHU protein binds to double stranded DNA

**Figure 9.**
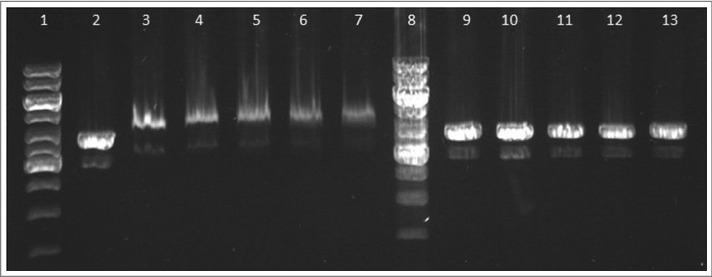
FtHU binds to dsDNA. Gel electrophoresis of FtHU-DNA complex. Lanes 1 and 8 standards, lane 2 naked DNA, lanes 3,4,5,6, and 7 DNA with FtHU (0.5; 0.7; 0.9; 1; 1.2 μg), lanes 9,10,11,12,and 13 DNA with BSA (0.5; 0.7; 0.9; 1; 1.2 μg). FtHU-DNA complex migrates slower in agarose gel and it is localized higher than control sample with BSA.

Electrophoretic mobility shift assay was used for detection of binding between FtHU and DNA. In that assay, we showed that FtHU forms binding complex with DNA. This FtHU-DNA complex is larger than naked DNA and migrates slower therefore it is localized higher on agarose gel. In the control samples, BSA protein has no DNA binding capacity, so there is no protein-DNA complex (Fig. 9).

### FtHU protein protects DNA from oxidative damage

**Figure 10.**
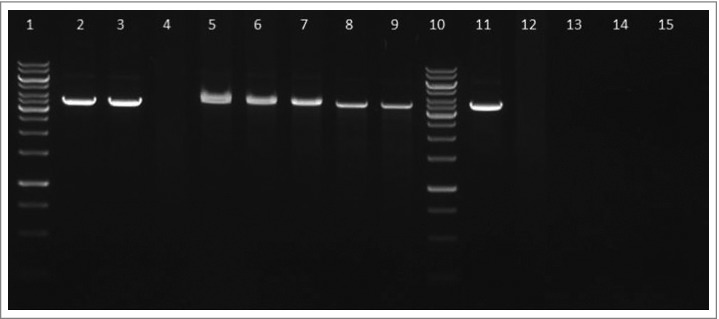
FtHU protects DNA from oxidative damage. The ability of FtHU to protect DNA from free hydroxyl radicals was tested. Samples were analyzed on 1% agarose gel and visualized by SYBR®Safe DNA gel stain. BSA protein was used as a negative control. 1 and 10 standards, 2 DNA, 3 DNA + H_2_O_2_, 4 DNA + H_2_O_2_ + Fe^2+^ (166 μM), 5–9 DNA + H_2_O_2_ + FtHU + Fe^2+^ (0 μM, 166 μM, 333 μM, 666 μM, 1000 μM), 11–15 DNA + H_2_O_2_ + BSA + Fe^2+^ (0 μM, 166 μM, 333 μM, 666 μM, 1000 μM). Even in case of increasing concentrations of Fe^2+^ HU is able to protect DNA from reactive oxygen species.

Next we performed DNA protection assay to found out if the FtHU is able to protect DNA against free hydroxyl radicals, which are generated via Fenton's reaction. Electrophoretic analysis was used for assessment of results of this assay. This study documented well the protective effect of FtHU on DNA. Even in case of increasing concentrations of Fe^2+^ protein is able to protect DNA from reactive oxygen species (Fig. 10).

## Discussion

Proper reaction to constantly changing environment is necessary part of life of intracellular pathogenic bacteria. To survive in fluctuating environment *Francisella* must have ability to regulate gene expression upon various external signals. Several transcription factors implicated in regulation of virulence and stress response genes have been described e.g. MglA /SspA (26–28), PigR [27,29–31], PmrA [32–34]. To date several different models have been suggested for how the expression of FPI genes is regulated [27,34–36]. Here, we demonstrated that HU protein is another key player that cannot be neglected.

As expected, we proved that *F. tularensis* HU protein has a capacity to bind to dsDNA and to protect DNA against free hydroxyl radicals, one of the main host defense tools. This is known for eukaryotic histones [37,38] and for prokaryotic histone-like proteins as well [39,40]. The ability of HU protein to protect DNA is often connected with the bacterial resistance to oxidative stress. This is also well documented in our study; the *F. tularensis* FSC200/*hupB* mutant strain is much more sensitive to the CuCl_2_- and H_2_O_2_-induced oxidative stress conditions than WT strain. This is in agreement with studies on other bacterial species lacking functional HU protein [40,41]. *F. tularensis* as an intracellular pathogen needs to efficiently cope with oxidative stress induced by reactive oxygen species in the intracellular niche. Thus, the inability of the FSC200/*hupB* mutant strain to persist in the conditions of oxidative stress might contribute to reduced replication inside murine macrophages and *in vivo* attenuation, as well.

Systematic transcriptomic analysis of *E. coli* gene expression in the absence of one or both HU subunits revealed changes in 8% of *E. coli* genome [14] including genes responsible for anaerobiosis, acid stress, high osmolarity, and SOS induction. We examined the possible role of HU protein in *Francisella* gene expression using iTRAQ analysis. This investigation revealed that the quantities of more than 400 different proteins were influenced by the loss of the HU protein. Additionally, this proteomic study clearly documented that the FSC200/*hupB* mutant attenuation is complex process. With respect to oxidative stress response several proteins were found to exhibit decreased synthesis in the *hupB* null mutant. Of them e.g. thioredoxin that is associated with a number of proteins involved in oxidative stress response like superoxide dismutase, hydrogen peroxidase I, alkyl hydroperoxide reductase was identified. Interestingly, the HU protein was recently detected as thioredoxin partner in *E. coli* [42]. Other proteins with decreased expression related to redox homeostasis encompassed glutaredoxin and SodB superoxide dismutase. Glutaredoxin together with thioredoxin is known for maintaining a cellular reducing environment [43] and SodB makes the conversion of superoxide anion to hydrogen peroxide in bacterial periplasm during phagocytosis [44].

The FPI proteins, with the exception for IglG, represent another group of virulence factors whose production is dependent on the functional HU protein. Especially, IglABCDEJIH, VgrG, PdpABCE and DotU that are chiefly responsible for escape of bacteria to the cytoplasm and their intracellular survival [45] and are involved in the assembly of type six secretion system of *Francisella*, too [46–48]. So far, it was believed that the expression of FPI proteins is strictly dependent upon action of three transcription regulator called MglA, SspA and PigR [26–29]. The former two form a heterocomplex which associate with RNA polymerase [27], the latter one is DNA-binding protein that interacts with MglA/SspA-complex. The protein levels of MglA and SspA detected in the FSC200/*hupB* mutant and WT did not significantly differ (Table S1). MglA/SspA regulatory complex works together with a DNA binding protein PigR [29] and this interaction is promoted by the alarmone guanosine tetraphosphate (ppGpp) that is controlled by *spoT* and *relA*, depending on variety of stress signals [35]. In course of manuscript preparation Cuthbert *et al* [36]. published study which revealed the direct link between sensing of ppGpp molecule and activation of FPI transcription via MglA/SspA/PigR/ppGpp machinery. Interestingly, RelA did not show significant proteomic change in *hup*B null mutant, on the other hand the SpoT exhibits significantly increased synthesis (1.36 fold change). Thus the amount of ppGpp in cell should be sufficient. Protein PigR did not fulfill the criteria set for evaluation of the iTRAQ data, nevertheless its quantification by targeted proteomics revealed its significantly decreased synthesis. Likewise PigR, the IglG protein, the only FPI protein not identified in iTRAQ analysis, exhibited significantly decreased synthesis in the FSC200/*hupB* mutant strain by targeted proteomic approach. These proteomic data were further confirmed on the transcription level. Semi-quantitative RT-PCR demonstrated that expression of *pigR* and FPI genes is negatively altered in *hupB* null mutant. Taken together, our findings thus strongly suggest the involvement of HU protein in regulation of *pigR* and thus FPI genes expressions and make it an important component of *Francisella* virulence.

Surface polysaccharides, including lipopolysaccharide (LPS) and possibly a capsular polysaccharide, have been proposed as important *Francisella* virulence determinants [49,50]. The LPS of *F. tularensis* is similar to that of other gram-negative bacteria [51], however, unlike the LPSs of most other pathogenic bacteria, the LPS of *F. tularensis* is considered nontoxic because it does not induce a pro-inflammatory cytokine response [52]. We identified 10 from 15 of *wbt* gene cluster products, implicated in O-antigen biosynthesis, with decreased synthesis in *hupB* null mutant strain (namely WbtABCEFHIJKL). Interestingly, when comparing the O-antigen laddering it seems that some O-antigen subunit might be missing in *hupB* null mutant, and the global amount of O-antigen seems to be affected as well. This is in agreement with the results of Priyadarshini *et al* [12]. that demonstrated role of Hu-β in regulation of LPS genes transcription in *P.  gingivalis*.

Generally, strains lacking the O-antigen are highly sensitive to killing by serum complement, less resistant to the host antimicrobial peptides and antibodies [53]. Our results are in accordance with this observation because the FSC200/*hupB* mutant exerts an increased sensitivity to human serum comparable to FSC200/*wbtDEF::Cm*, which lacks O-antigen. Noteworthy, mutants which lack LPS are severely attenuated or avirulent but fail to elicit a protective immune response [54,55]. Similarly as in case of FTS_1402 mutant [24] the *hupB* null mutant that retains O-antigen protects mice against WT challenge. This is in strong agreement with hypothesis that the presence of LPS even with altered structure or reduced quantity is necessary for providing protection against subsequent WT challenge.

Clue about how the HU protein is activated or what exact role it plays needs to be further addressed. Gupta *et al* [56]. proved that in *Mycobacterium* HU protein is substrate for phosphorylation by serine/threonine kinases and thus it could act as a signal molecule. Another post-translational modification, acetylation, of mycobacterial HU protein could lead to activation of gene expression and protein-protein interaction [57]. Also the manner how it protects DNA or what binding sites on double stranded DNA it recognizes, have not been uncovered yet.

To conclude, the results of this study highlight the importance of HU protein in *Francisella* virulence. Our both *in vitro* and *in vivo* assays showed that *Francisella* mutant strain lacking *hupB* cannot resist to murine macrophages and is not able to cause tularemia in mice. Not only those mice survived mutant strain infection, but also the protective effect against WT challenge was proved. Next, we confirmed the ability of the FtHU to bind to dsDNA and protect dsDNA against free hydroxyl radical damage. Moreover, proteomic analysis of mutant strain showed a battery of differently expressed proteins, which are involved in stress response, transcription, cell division, etc. Strikingly, the decreased synthesis of the complete set of proteins encoded within the FPI as well as several enzymes participating in the O-antigen biosynthesis was observed too. The quantitative proteomics then revealed that diminished expression of the PigR transcription factor might be responsible for the defect in the FPI proteins production. Globally the obtained results further extend the current knowledge of the *Francisella* virulence mechanisms and might contribute to the development of a novel strategy for effective treatment of tularemia disease.

## Materials and methods

### Bacterial strains, plasmids and growth conditions

Bacterial strains and plasmids used in this study are listed in Table S1. *Francisella* strains were cultured on McLeod agar plate enriched for bovine hemoglobin (BD Diagnostics, 212392) and IsoVitalex (BD Diagnostics, 211876) or in Chamberlain medium [58] with shaking 200 rpm at 37°C. The *E. coli* strains were cultured in Luria Bertani (LB) broth medium or on LB agar plates. When necessary antibiotics were used at following concentration: kanamycin 20 μg/ml (*F. tularensis*) or 50 μg/ml (*E. coli*), ampicillin 100 μg/ml (*E. coli*).

### Construction of a *hupB* null mutant strain

*Francisella hupB* null mutant strain was prepared by allelic exchange. The *hupB* deletion construct was amplified by overlapping PCR using primers A, B, C and D, described in Table S2. This construct was cloned into pCR4-TOPO TA cloning vector (Invitrogen, 450030) to facilitate sequencing (Institute of Microbiology, CAS). Then the deletion construct was cut out using *Xho*I/*Sac*I restriction sites and ligated into suicide pDM4 vector using the same restriction sites. Conjugal mating between *E. coli* S17–1 λpir donor strain and *F. tularensis* subsp. *holarctica* strain FSC200 followed by sucrose-selection led to the allelic exchange on the FSC200 chromosome. PCR screening with primers 1F, 2R, F1 and R1 (Table S2) was used to verify the *hupB* deletion in the obtained transformants.

### Complementation *in trans*

*Francisella hupB* was amplified by PCR using pKK_0886_F and pKK_0886_R primers (Table S2) and cloned into pCR4-TOPO TA cloning vector (Invitrogen, 450030) to facilitate sequencing. Using *Sac*I and *Nde*I endonucleases the *hupB* was excised from pCR4-TOPO TA vector and ligated to *Nde*I/*Sac*I-digested pKK289KmGFP shuttle vector. The final construct pKK289Km-*hupB* was introduced into *F. tularensis* subsp. *holarctica* FSC200/*hupB* by electroporation [59]. The complemented mutant was denoted as FSC200/*hupB+hupB*.

### Cloning of recombinant protein FtHU/FSC200

The *hupB* was amplified using primers pET_rHuB_F and pET_rHuB_R (Table S2) and cloned into the pCR4-TOPO TA cloning vector (Invitrogen, 450030). Following sequencing the *hupB* fragment was cut out using *Nco*I and *Xho*I restriction sites and cloned under the T7 promoter of pET28b (+) expression vector (Novagen, 69865–3). Plasmid pET28b-*hupB* was introduced into *E. coli* BL21 (DE3) (Novagen, 69450) allowing overexpression of the FtHU protein.

### Purification of FtHU

*E. coli* BL21 FtHU strain was grown in LB medium with appropriate antibiotic overnight. Next day the culture was diluted in 50 ml and was cultivated to reach the optical density at 600 nm of 0.5. Then the culture was poured into 500 ml TB ON medium (Merck Millipore, 71491–5) with antibiotic and was cultivated 8 hours at 30°C. Culture was centrifuged at 8500 rpm, 4°C, and 10 min. The pellet was washed with pre chilled Tris buffer (pH 8). The pellet was resuspended in 10 ml PBS with 350 mM NaCl, protease inhibitors (Roche, 05056489001) and benzonase nuclease (Sigma Aldrich, E1014). Cell lysate was prepared by french press (16 000 psi).

Purification of FtHU was done with Amicon Pro Affinity Concentrator (Merck Millipore, ACK5003PG) and HisLink Protein Purification Resin (Promega, V882A). The sample was mixed with resin and incubated at 4°C for 30 min. Proteins unbound to the resin were washed out two times by Lysis buffer (50 mM sodium phosphate buffer, 300 mM NaCl, 10 mM imidazole) and once by Wash buffer (50 mM sodium phosphate buffer, 300 mM NaCl, 20 mM imidazole). FtHU was eluted from the resin using Elution buffer (50 mM sodium phosphate buffer, 300 mM NaCl, 250 mM imidazole). The imidazole was removed from the sample by PD MiniTrapG-25 (GE Healthcare Life Sciences, 28–9180–07). The sample was concentrated with Amicon Ultra 3K (Merck Milipore, UFC800324). Presence of FtHU was determined by SDS-PAGE. Concentration of protein was calculated by BCA assay (Thermo Fisher Scientific, 23225). Purity of HU protein used in this experiment is well documented (Fig. S5).

### Isolation of macrophages and *in vitro* proliferation

Bone mouse marrow cells were isolated from femur of 6–10 weeks old female BALB/c mice (Velaz, Unetice, Czech Republic). The cavity of the femur was flushed out with DMEM (Sigma Aldrich, D0819) and bone marrow cells were collected. Suspension was centrifuged (5 min, 400 x g, RT) and the cells were resuspended in BMMs medium (DMEM supplemented with 10% fetal bovine serum (FBS, Dominique Dutscher, S181H-500) and 10% L929-conditioned medium) with appropriate antibiotics (50 U/ml penicillin, 50 μg/ml streptomycin; Sigma Aldrich, P0781). The cells were incubated in concentration of 6 × 10^6^ per Petri dish for one week to differentiate to bone marrow-derived macrophages (BMMs). The day before infection, macrophages were seeded on tissue culture plates in concentration of 5 × 10^5^ per well. Following cultivation overnight, BMMs were infected by all three bacterial strains (FSC200, FSC200/*hupB* and FSC200/*hupB+hupB*) at a multiplicity of infection (MOI) of 50. Infection started by centrifugation of plates for 5 min, 400 x g, and then the samples were incubated at 37°C, 5% CO_2_ for 30 min. Extracellular bacteria were killed during incubation in DMEM with 5 μg/ml gentamicin for 30 min at 37°C, 5% CO_2_. The cells were washed three times with preheated PBS before the DMEM with 10% FBS without antibiotics was added. At particular time points (1, 6, 24 and 48 hours) after infection cells were lysed by 0.1% sodium deoxycholate. The lysates were plated on McLeod agar plates in appropriate dilution. The plates were incubated at 37°C, 5% CO_2_ for several days. The number of viable intracellular bacteria was determined by colony forming units (CFU) counting.

### Infection of mice

Groups of five female BALB/c mice, 6–10 weeks old, were infected with 200 μl of bacterial suspension subcutaneously (s.c.) with appropriate infection doses of 10^1^, 10^2^, 10^3^, 10^4^, 10^5^ and 10^6^ CFU/mouse for FSC200/*hupB* mutant and 10^2^ CFU/mouse for FSC200 and complemented strain FSC200/*hupB+hupB*. Control group of mice was inoculated with physiological saline solution. Mice were observed for 21 days and eventually deaths were noted. The survivors were challenged subcutaneously 30 days post infection with 10^2^ CFU/mouse of the FSC200 to study the ability of the mutant strain to protect mice against WT challenge.

### Standard and stress growth kinetics

Bacterial strains (FSC200, FSC200/*hupB*, FSC200/*hupB+hupB*) were grown in Chamberlain medium with appropriate antibiotics at 37°C, 200 rpm overnight. The cultures were diluted to a final optical density at 600 nm of 0.1 in complete Chamberlain medium (for standard growth curve) and in Chamberlain medium supplemented with CuCl_2_ (Sigma Aldrich, 222011) to a final concentration of 20 μM (for oxidative stress growth curve). 200 μl aliquots of the suspensions were applied to a 96-well plate in pentaplicates. Remaining wells were filled with water to prevent evaporation. The growth kinetics was determined by measurement of optical density at 600 nm using microplate reader FLUOstar Optima (BMG Labtech, Offenburg, Germany). Experiment was repeated three times.

### Oxidative stress survival assay

Bacterial strains (FSC200, FSC200/*hupB*, FSC200/*hupB+hupB*) were grown in Chamberlain medium with appropriate antibiotics at 37°C, 200 rpm overnight. Samples were diluted to an optical density at 600 nm of 0.1 in complete Chamberlain´s medium. The cultures were incubated at 37°C, 5% CO_2_ and 200 rpm to reach final optical density of 1–1.2. Hydrogen peroxide was added to a final concentration of 0.03% to each flask. At selected time points (1, 2, 3 and 4 hours after H_2_O_2_ addition) the cultures were serially diluted and plated on McLeod agar plate (with appropriate antibiotics). The growth kinetics in oxidative stress environment was determined by CFU counting.

### Electrophoretic mobility shift assay (EMSA)

EMSA was performed with purified recombinant *Francisella* HU protein (FtHU) in different amounts (0.5 – 1.2 μg) and with 500 ng of linearized plasmid DNA (pBluescriptSK+, Stratagene, 212205) in final volume of 10 μl. The reaction was performed in binding buffer (20 mM Tris-HCl, pH 8, 0.1 mM EDTA-Na_2_, 50 mM KCl, 10 μg/ml BSA, 5% glycerol, 0.1 mM DTT, 0.05% Brij 58) for 20 minutes on ice. Then the whole sample was loaded onto a 0.5% agarose gel, using 0.33x TBE buffer. Bovine serum albumin (BSA, Sigma Aldrich, P0914) was used as negative control. Electrophoretic separation was run at 4°C, 50 V for 5 hours. The gel was visualized by SYBR®Safe DNA gel stain (Invitrogen, S33102).

### DNA protection assay

The ability of FtHU (0.5 μg) to protect 500 ng of linearized plasmid DNA (pBluescriptSK+, Stratagene, 212205) from free hydroxyl radicals was tested in the binding buffer (20 mM Tris-HCl, pH 8, 0.1 mM EDTA-Na_2_, 50 mM KCl, 10 μg/ml BSA, 5% glycerol, 0.1 mM DTT, 0.05% Brij 58) in a total volume of 10 μl. After 30 min incubation at RT, both H_2_O_2_ (final concentration of 10 mM) and Fe^2+^ (final concentration of 0; 166; 333; 666 and 1000 μM) were added. Samples were incubated for further 15 min at 37°C. The DNA-protein bond was disrupted by incubation at 85°C for 5 min. Samples were analyzed on 1% agarose gel (100 V, 35 min) and visualized by SYBR®Safe DNA gel stain (Invitrogen, S33102). BSA protein was used as a negative control.

### iTRAQ analysis

#### iTRAQ sample preparation

The pellets of WT and mutant strain, obtained from Chamberlain medium culture OD_600_ 0.6–0.7, were washed with pre chilled PBS, suspend in water. 4% sodium deoxycholate was added in 1:1 ratio and the bacteria were lysed at 70 °C for 3 min. DNA was cleaved by sonication. Protein concentration was determined using a BCA assay (Thermo Fisher Scientific, 23225). A total of 25 μg of each sample was reduced with tris(2-carboxyethyl)phosphine hydrochloride (TCEP), alkylated with methyl methanethiosulfonate and digested with trypsin (Promega, V5111). Peptides were dried and re-dissolved in triethylammonium bicarbonate buffer pH 8.5. Samples were labeled with iTRAQ reagents (Applied Biosystems, 4352135) for 5 hours at room temperature. Each sample was labeled with two of four available iTRAQ tags. Labeled peptides were mixed to the two sets, where both contained each of the iTRAQ tags (Table S3). Water saturated ethyl acetate (EA) was added to each sample to extract deoxycholate. The samples were acidified with HCl to pH of 1.5 and washed three times with EA. After adding water, the peptides were dried desalted using Empore C18-SD (Sigma-Aldrich, 66871-U) cartridges and dried in a vacuum centrifuge.

#### High pH reversed-phase peptide fractionation

High pH reversed-phase peptide fractionation was performed using a modified microgradient device described previously [60,61]. Peptides were re-dissolved in 20 mM NH_4_FA in 2 % acetonitrile, pH 10 and injected onto a prepared microcolumn in a FEP tubing (length 34 mm, 250 µm i.d.), packed with Kinetex EVO C18 2.6 µm core-shell particles (Phenomenex, 00G-4725-E0). The peptides were eluted by an acetonitrile gradient ranging from 2% to 40% all in 20 mM NH_4_FA, pH 10, over the elution volume of 24 µl. The eluted peptides were collected in 14 fractions. Fractions were acidified with 1% TFA, dried in a vacuum centrifuge and reconstituted in 30 µl of 2% ACN, 98% water and 0.05% TFA. Fractions were then combined into seven samples for LC-MS analysis 1+7, 2+8, 3+9, 4+10, 5+11, 6+12, 13+14.

#### LC-MS/MS analysis of iTRAQ labeled peptides

UltiMate 3000 RSLCnano system (Dionex, Sunnyvale, USA) hyphenated through Nanospray Flex ion source to Q-Exactive mass spectrometer (Thermo Fisher Scientific, Waltham, USA) were used for protein identification and quantification. Peptides were loaded on PepMap100 C18, 3 µm, 100 Å, 0.075 × 20 mm trap column and separated on PepMap RSLC C18, 2 µm, 100 Å, 0.075 × 150 mm analytical column by a gradient formed by mobile phase A (0.1% FA) and mobile phase B (80% ACN, 0.1% FA), running from 4 to 34% in 48 min, and from 34 to 55% of mobile phase B in 15 min at a flow rate of 0.3 µl/min at 40 °C. The full MS/Top10 experimental setup was used. Positive ion full scan MS spectra (*m*/*z* 375–1650) were acquired using a 1 × 10^6^ AGC target at resolution 35.000 (at *m*/*z* 200) and a maximum 50 ms injection time. Precursors of charge state ≥ 2 were selected for HCD fragmentation, with an exclusion window of 30 s. The isolation window of 1.6 Da and normalized collision energy of 28 was used. Each HCD spectrum was acquired at resolution 35.000 with a 1 × 10^5^ AGC target and a maximum 150 ms injection time.

#### iTRAQ data evaluation

HCD spectra were searched in Proteome Discoverer software (Thermo Fisher Scientific, Waltham, USA) using MASCOT engine (Matrix Science, London, UK), first with a mass tolerance of 7.5 ppm for precursors and 10 mmu for fragments against sequence of the porcine trypsin. A maximum of two missed cleavages were allowed. Methylation and dimethylation of Lys, oxidized Met and deamidated Asn were set as dynamic modifications, while thiomethylation of Cys, iTRAQ modification of Lys and peptides N-terminus were set as fixed. Spectra identified with FDR > 0.01 were subjected to a secondary search against a *Francisella tularensis* subsp. *holarctica* database. The tryptic specificity was set to full and two missed cleavages were allowed. The mass tolerance was set to 12.5 ppm for precursors and 20 mmu for fragment ions. Oxidized Met and N-terminal acetylation were set as dynamic modifications, while thiomethylation of Cys, iTRAQ modification of Lys and peptide N-terminus were set as fixed modifications. Percolator was used for rescoring MASCOT search results. Identifications up to 1% FDR were reported. Only precursors with a maximum 20% co-isolation, resulting in identification of unique peptides were used for quantification. Quantitative results were corrected on purity factors and normalized on protein median. Only proteins quantified with at least two peptides in both iTRAQ sets were evaluated. For each protein, four iTRAQ log_2_ ratios to the WT channels were tested for difference using one-sample *t*-test. False discovery rate due to the multiple hypothesis testing was controlled using Benjamini-Hochberg method at α = 0.05.

### Targeted quantification of proteins using LC-PRM (liquid chromatography-parallel reaction monitoring)

For a maximum accuracy of the relative LC-PRM quantification, a *F. tularensis* FSC200 heavy culture was prepared. Bacteria were inoculated into Chamberlain defined medium in which ordinary Arg and Lys were substituted by amino acids labeled with heavy stable isotopes: Arg [^13^C_6_^15^N_4_] (Sigma Aldrich, 608033) and Lys [^13^C_6_] (Sigma Aldrich, 643459) and cultivated overnight. This culture was aliquoted and kept at -80°C. For the final experiment, an aliquot was inoculated into the heavy medium and cultivated overnight. The overnight culture was diluted into heavy medium to OD_600_ = 0.1 and grown until the optical density 0.6. This culture was processed as described in the section “iTRAQ sample preparation”. An LC-MS/MS analysis confirmed total labeling of the bacterial proteins with the heavy amino acids (data not shown).

The LC-PRM assay development, including method refinement and final data analysis were all performed in a Skyline software (University of Washington, Seattle, WA) [62]. First of all, an MS/MS library of *F. tularensis* FSC200 tryptic peptides was built to facilitate and accelerate PRM method design [63]. FASTA sequences (with and without the initiator Met) of proteins of interest i.e. PigR and IglG were imported into the Skyline software and following criteria were applied on peptide selection: digestion – trypsin; no missed cleavage allowed; peptide length – between 6 and 20 amino acids; structural modifications – carbamidomethylation at Cys. Doubly and triply charged precursors were considered. Only peptides and precursors matching the criteria and present in the MS/MS library were allowable. Two peptides were found for PigR protein (ANQYSGNFEQIVK and EGLSYQDAEK) and one peptide was found for IglG (TLTNNLPASNITDSK). In addition, two peptides for HU protein (SELVSAIAK and EVASNTLDATIAAVTK) were added to ultimately confirm deletion of its gene from the chromosome. An isolation list for these peptides containing *m*/*z* of precursors along with *m*/*z* of labeled counterparts was imported into a Q-Exactive PRM method. MS2 spectra of each precursor were recorded continuously within five minutes of previously confirmed retention time using a 2 × 10^5^ AGC target at resolution 35.000 (at *m*/*z* 200) and a maximum 125 ms injection time. The isolation window of 1.8 Da and normalized collision energy of 27 was used.

For final quantification, the heavy culture lysate (10 μg) was added to both, FSC200 and FSC200/*hupB* strain lysates (10 μg) in quadruplicates. Proteins were then reduced with TCEP, alkylated with iodoacetamide and before digestion with trypsin; the excess of iodoacetamide was quenched by adding of cysteine. The same instrumentation as specified in section “LC-MS/MS analysis of iTRAQ labeled peptides” was used for recording the PRM data during the same gradient as used for separation of iTRAQ peptides. From the recorded data, Skyline software calculated peak areas for 3 most intense fragments with *m*/*z* from (*m*/*z* precursor – 1) observed in the MS2 spectra and normalized to those recorded from the heavy precursors. Normalized peptide levels in FSC200 and FSC200/*hupB* strains were log_2_ transformed for statistical evaluation.

### Semi-quantitative analysis of *pigR* and FPI genes expression

RNA from WT, complemented and mutant strains was isolated from Chamberlain medium cultures of OD_600_ 0.7 using RNeasy Mini Kit (Qiagen, 74106) according to the manufacturer's instruction. Obtained RNA was treated with DNase I (Invitrogen, 18068–015). Aliquots of RNA were used for reverse transcription and the obtained cDNA was used for PCR amplification of target genes using appropriate primers (Table S5). Samples were analyzed by gel electrophoresis and intensities of bands were determined by Bio1D software (CertainTech, Sterling, VA, USA). Normalized transcription levels in all three strains were plotted to the graph.

### Purification of LPS

Bacteria were grown in a Chamberlain medium at 37 °C, 200 rpm until the late logarithmic phase of growth. Following the lysis of bacteria in a French pressure cell, a membrane pellet was obtained by ultracentrifugation of the whole-cell lysate and suspended in PBS. Protein content was determined using BCA assay. Further, LPS was extracted from 0.5 mg of a membrane proteins-enriched fraction using a hot phenol-water extraction method by Westphal and Jann with modifications [64]. Residual phenol was removed from the collected aqueous LPS-containing phase by acetone precipitation prior to polyacrylamide gel electrophoresis using sodium dodecyl sulfate (SDS-PAGE).

### SDS-PAGE, immunodetection and LPS staining

Purified recombinant protein and isolated LPS were separated on a one-dimensional SDS-PAGE and electroblotted onto PVDF membranes. FtHU and LPS were detected using a polyclononal rabbit anti FTS_0886 serum (Moravian Biotechnology) and a mouse monoclonal anti-LPS FB11 antibody (Abcam, AB2033), respectively. As secondary antibodies the polyclonal swine antirabbit IgG/HRP (Dako, P0399) and polyclonal goat antimouse IgG/HRP (Dako, P0447) were used. Chemiluminescence detection was employed using a BM Chemiluminescence Blotting Substrate (POD) while following the manufacturer's instructions (Roche, 11500694001). LPS staining was done using Pro-Q Emerald 300 Gel Stain kit (Invitrogen, P20495). Images of gels were collected using a UV transilluminator (Vilber Lourmat, Eberhardzell, Germany).

### Serum sensitivity assay

Bactericidal assay was conducted with fresh sera prepared from the whole blood of anonymous healthy nonimmune volunteers. Briefly, isolated nonheparinized whole blood was kept at room temperature for 1 h and then for 30 min at 4°C to allow for clot formation and clot contraction, respectively. The clot was removed by centrifugation at 500  ×  g for 30 min at 4°C. The serum fraction was collected, centrifuged at 500  ×  g for 5 min, aliquoted, and stored at −80°C until needed but no longer than 3 weeks. Bacteria were transferred from the McLeod agar plates to PBS and the suspensions of OD_600_  =  1 were prepared. Suspension volumes corresponding to 5  ×  10^7^ bacteria were diluted in a 1 ml of PBS. For each assay, 40 μl of diluted bacterial suspensions containing 2  ×  10^6^ bacteria were added to 160 μl of 100% serum (final concentration of 80%), or 6.25% serum in PBS (final concentration of 5%), and the mixtures were incubated for 90 min at 37°C. As a positive control, 160 μl of PBS was used (100% survival). Lysis was stopped by incubation of tubes on ice for 5 min. Surviving bacteria were enumerated by plating 10-fold serial dilutions of each suspension. Experiment was performed in a triplicate.

### Ethics statement

All experiments using mice were performed in accordance with guidelines of the Animal Care and Use Ethical Committee of the Faculty of Military Heath Sciences, University of Defense, Czech Republic. The research protocol was approved by the ethics committee under project no. 50–6/2016–684800. Experiments using human sera were conducted with the approval of Ethics Committee of University Hospital Hradec Kralove; reference no. 201710S10P and each volunteer provided written informed consent to participate in this study in accordance with regulatory guidelines.

### Statistical analysis

Statistical significances were analyzed by GraphPad Prism version 5 (GraphPad Software, La Jolla, CA, USA). The degree of significance was defined using two-way ANOVA followed by Bonferroni's multiple comparisons test unless otherwise indicated. In statistical analysis FSC200/*hupB* mutant strain was compared to FSC200 strain. Statistical significance of targeted PRM data was tested using Student's t-test. P value < 0.05 *, P < 0.01 **, *P* < 0.001 ***, *P* < 0.0001 ****.

## Supplementary Material

1441588.pdf

## List of abbreviation

ACN: acetonitrile
AGC: automatic gain control
BCA: bicinchoninic acid
BMMs: bone marrow-derived macrophages
BSA: bovine serum albumin
CFU: colony forming units
Da: Dalton
DMEM: Dulbecco's modified eagle medium
DNA: deoxyribonucleic acid
EA: ethyl acetate
FBS: fetal bovine serum
FDR: false discovery rate
FPI: *Francisella* pathogenicity island
FSC: *Francisella* strain collection
FtHU: *F. tularensis* HU protein
H_2_O_2_: hydrogen peroxide
HCD: higher-energy collisional dissociation
HRP: horseradish peroxidase
iTRAQ: isobaric tags for relative and absolute quantitation
LB: Luria Bertani medium
LC-MS/MS: liquid chromatography tandem mass spectrometry
LC-PRM: liquid chromatography-parallel reaction monitoring
LPS: lipopolysaccharide
mmu: millimass unit
MOI: multiplicity of infection
ms: millisecond
MS2: fragmentation spectra
OD: optical density
PBS: phosphate buffered saline
ppGpp: guanosine tetraphosphate
ppm: parts per million
PVDF: polyvinylidene fluoride
RT: room temperature
s.c.: subcutaneous route of infection
SDS-PAGE: sodium dodecyl sulfate polyacrylamide gel electrophoresis
TB ON: Overnight express terrific broth medium
TCEP: tris(2-carboxyethyl)phosphine hydrochloride
TFA: trifluoroacetic acid
UV: ultraviolet
WT: wild-type
