# Supplementary material for: HU protein is involved in intracellular growth and full virulence of Francisella tularensis
Source: Virulence. 2018 Feb 23;9(1):754–70. doi: 10.1080/21505594.2018.1441588 (PMC5955460; doi:10.1080/21505594.2018.1441588)
Supplement: 1441588.pdf [file kvir-09-01-1441588-s001.pdf]

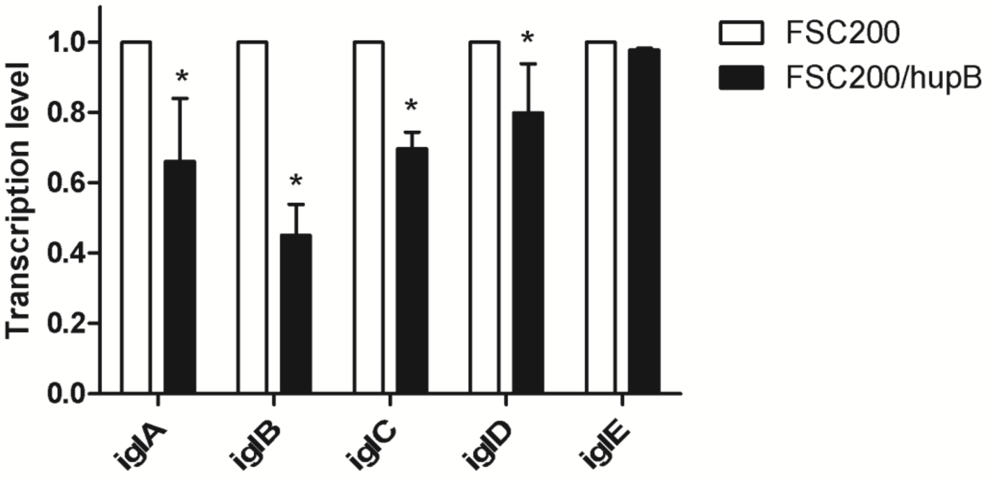
Transcription level
1.0
0.8
0.6
0.4
0.2
0.0
iglA
iglB
iglC
iglD
iglE
FSC200
FSC200/hupB
*

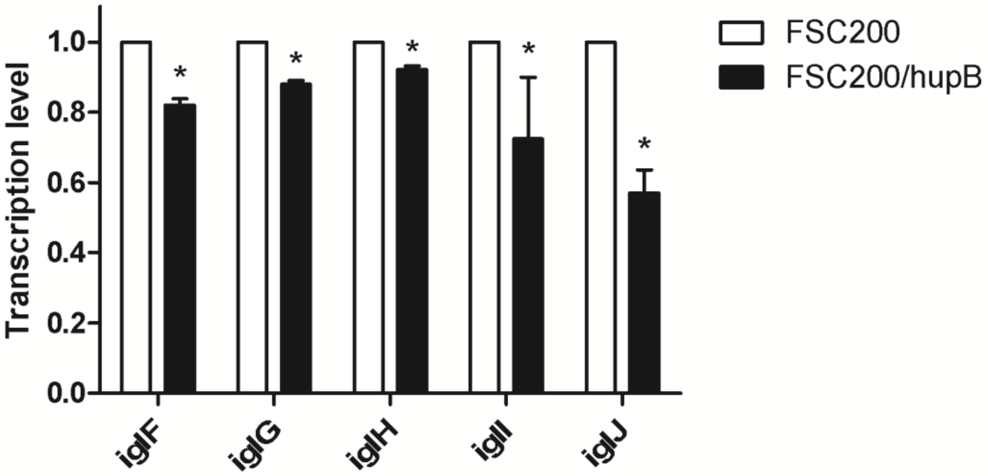
Transcription level
1.0
0.8
0.6
0.4
0.2
0.0
iglF
iglG
iglH
iglI
iglJ
FSC200
FSC200/hupB
*

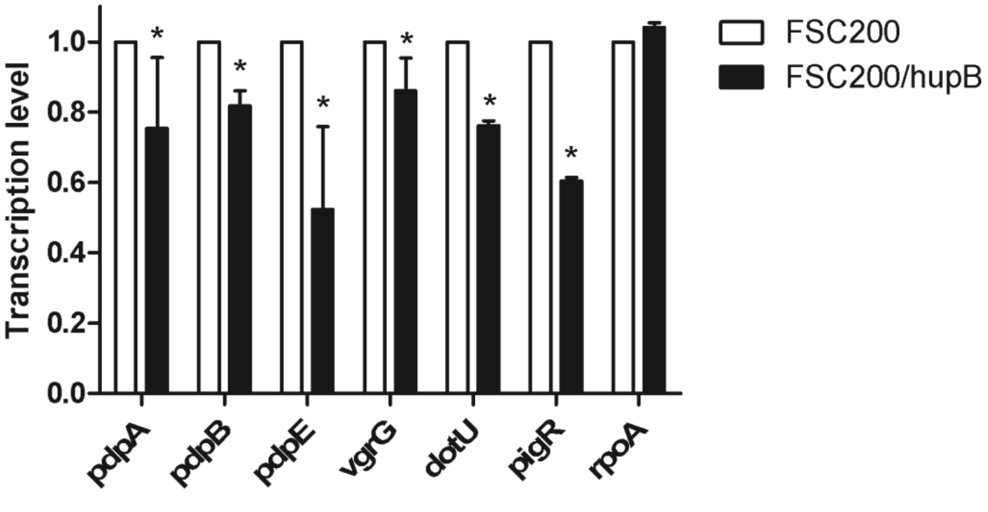
Transcription level
1.0
0.8
0.6
0.4
0.2
0.0
pdpA
pdpB
pdpE
vgrG
dotU
pigR
rpoA
FSC200
FSC200/hupB
*

Figure S1: Semi-quantitative RT-PCR of FPI genes. Reverse-transcription followed by PCR was used for verification of FPI production on transcription level. Almost all of FPI genes showed significantly decreased expression in mutant strain in contrast to WT, whereas the transcription level of *rpoA* remained unchanged. Taken together with iTRAQ data these results confirm role of HU protein in FPI expression. P value < 0.05 *, P < 0.01 **, P < 0.001 ***, P < 0.0001 ****.

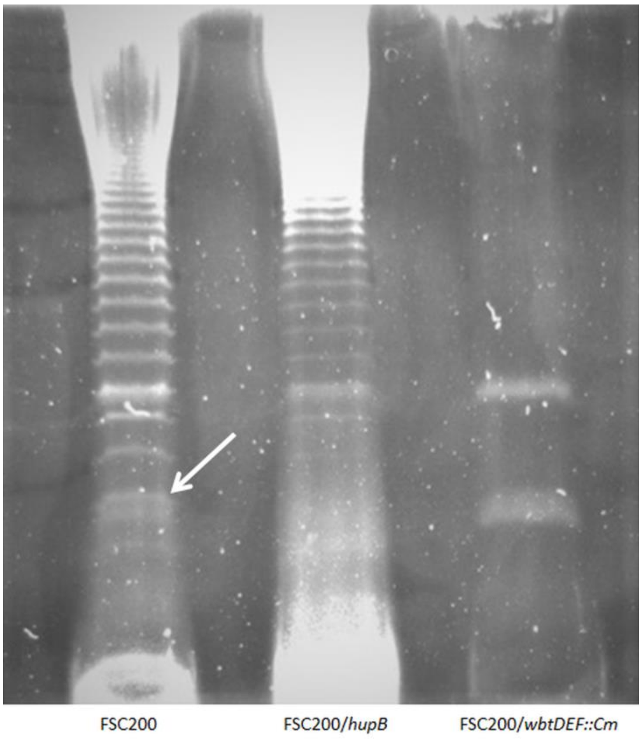

Figure S2: Pro-Q Emerald staining of LPS. LPS was extracted from the membrane proteins-enriched fractions using a hot phenol-water extraction. Residual phenol was removed from the collected aqueous LPS-containing phase by acetone precipitation prior to SDS-PAGE. For LPS visualization the Pro-Q Emerald 300 Gel staining was used. Strain FSC200/wbtDEF::Cm lacking LPS was used as a negative control.

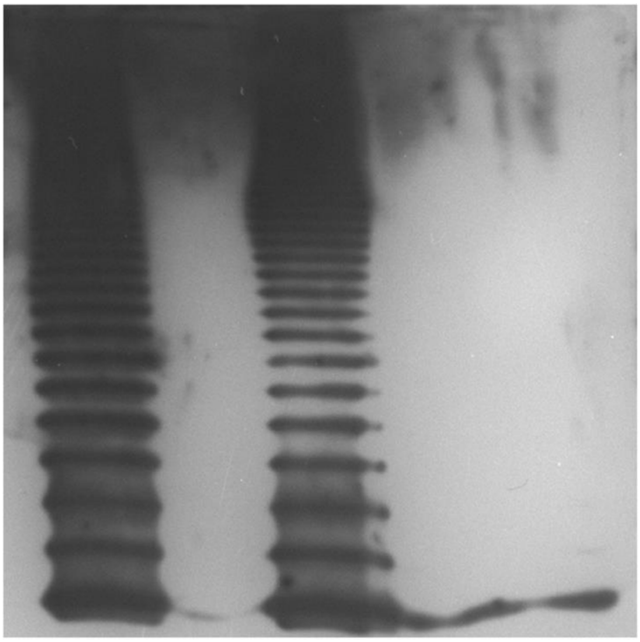

Figure S3: Western blot and immunodetection of LPS. Production of LPS was verified also by Western blot followed by immunodetection using anti-LPS antibody. *F. tularensis* subsp. *holarctica* FSC200/wbtDEF::Cm lacking LPS was used as a negative control. The typical ladder-like pattern of O-antigen was detected in the FSC200/*hupB* mutant but in comparison to WT strain with lower intensity in the middle part of the ladder.

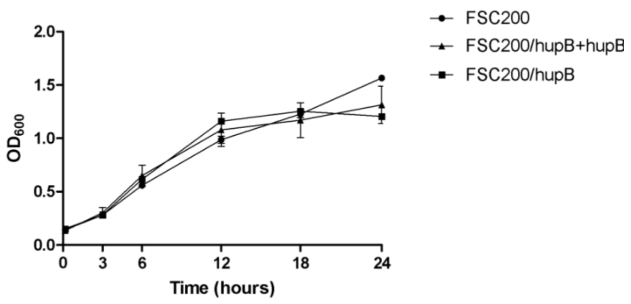

Figure S4: Standard growth curve. Bacteria were grown in Chamberlain medium with appropriate antibiotics at 37 °C for 24 hours in 96-well plate. The growth kinetics was determined by measurement of optical density at 600 nm using microplate reader FLUOstar Optima. Mutant strain exhibits very similar growth curve like WT and complemented strains.

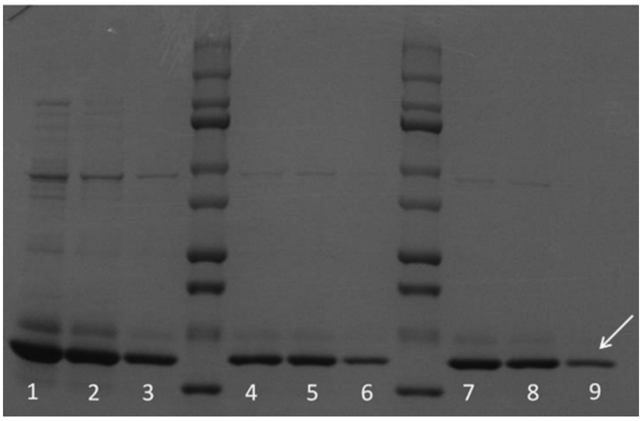

Figure S5: His-tagged HU protein purification. Purification of FtHU was done with Amicon Pro Affinity Concentrator and HisLink Protein Purification Resin. The samples were separated on 12% polyacrylamide gel. Samples 1-3 eluates, 4-6 eluates after imidazole removal (using PD MiniTrapG-25), 7-9 concentrated samples (using Amicon Ultra 3K). White arrow shows the position of FtHU.

TABLE S1 Bacterial strains and plasmids used in this study

Strain	Description	Source
Francisella tularensis FSC200	*Francisella tularensis* subsp. *holarctica*, wild type (WT), clinical isolate	*Francisella* Strain Collection (FSC) of the Swedish Defense Research Agency, Umeä, Sweden
FSC200/*hupB*	*hupB* deletion mutant strain, Δ*hupB*	This study
FSC200/*hupB*+*hupB*	deletion mutant strain complemented *in trans*	This study
FSC200/*wbtDEF::Cm*	FSC200 with inactivated O-antigen production	[65]
E. coli S17-1 λpir	*Escherichia coli* donor strain for conjugation TpR SmR recA, thi, pro, hsdR-M+RP4: 2-Tc:Mu: Km Tn7 λpir	[66]
E. coli XL1	*Escherichia coli* competent cell *recA1 endA1 gyrA96 thi-1 hsdR17 supE44 relA1 lac* [F′ *proAB lacIq Z*ΔM15 Tn*10* (Tet^R)]	Stratagene
E. coli BL21 (DE3)	*Escherichia coli* strain for overproduction F^- *ompT* $hsdS_B$ (r_B^- m_B^-) *gal dcm* (DE3)	Novagen
E. coli BL21/FtHU	*Escherichia coli* strain expressing recombinant *F. tularensis* subsp. *holarctica* FSC200 HU protein	This study

Plasmid	Description	Source
pBluescript SK+	cloning vector, f1 *ori*, *lacZ*, ColE *ori*, Amp^R	Invitrogen
pET28b	T7 expression vector, Km^R	Novagen
pCR4-TOPO	cloning vector, pUC *ori*, P_{lac}, *lacZ*, Kan^R, Amp^R	Invitrogen
pDM4	*F. tularensis* suicide vector, mob_{RP4},ori_{R6K}, *sacB*, Cm^R	[67]
pKK289KmGFP	*E. coli/ F. tularensis* shuttle vector, Ft *ori*, p15a *ori*, Km^R, groES promoter	[68]

TABLE S2 Primers used in this study[a]

Primer	Sequence	reverse/forward	Application
A	5´GCATGTCTCGAGTATGTGCGTATGGCTTT3´	F	deletion construct
B	5´ACTTTTTATTATTTTTCACTCTTGTTCATGTTTTTAAA3´	R	
C	5´ACAAGAGTGAAAAATAATAAAAAGTTACAAAAAAGTAA3´	F	
D	5´GCATGTGAGCTCTCTTATCTATCTTCTTTCCGCT3´	R	
1F	5´TGGGGTAAGAGGGCAAAAGT3´	F	PCR screening of mutant strain
2R	5´CTACTAGAGTTACGCTATCAC3´	R	
F1	5´AATGACAGGTGAGGTGACAC3´	F	
R1	5´CTCAAGTTTATCCATTCCACC3´	R	
pKK_0886_F	5´AAACATATGAACAAGAGTGAATTAGTAAG3´	F	complementation *in trans*
pKK_0886_R	5´AACGAGCTCTTATTTTACAGCGTCTTTAAGAC3´	R	
pET_rHuB_F	5´CCATGGCTAACAAGAGTGAATTAG3´	F	recombinant protein
pET_rHuB_R	5´CTCGAGTTTTACAGCGTCTTTAAGACC3´	R	

[a] The restriction sites are underlined.

TABLE S3 iTRAQ tags mixtures

iTRAQ	set 1	set 2
114	FSC200 (1)	FSC200 (3)
115	200/*hupB* (1)	200/*hupB* (3)
116	FSC200 (2)	FSC200 (4)
117	200/*hupB* (2)	200/*hupB* (4)

TABLE S5 Primers used in RT-PCR

Primer	Sequence	reverse/forward
iglA_F	5´CCGCGGAGCAAAAAATAAAATCCCAAATTCA3´	F
iglA_R	5´CTCGAGCTTACCATCTACTTGTTGATTA3´	R
iglB_F	5´ACAATAAATAAATTAAGTCTCACT3´	F
iglB_R	5´GTTATTATTTGTACCGAATAATTC3´	R
iglC_F	5´CCGCGGAAGTGAGATGATAACAAGACAAC3´	F
iglC_R	5´CTCGAGTGCAGCTGCAATATATCCTATT3´	R
iglD_F	5´CTCTTAATCATTATTATTTAGGTGAT3´	F
iglD_R	5´AGAAAAGGCTATAAAGAAATCAA3´	R
iglE_F	5´TACAATAAATTATTGAAAAATCTTTG3´	F
iglE_R	5´ATCTTTTTCTATGCTACTATCATT3´	R
iglF_F	5´AATAATAATATTGATAAATGGTTTGA3´	F
iglF_R	5´TCAGTACAATCTAAGAGGTTATC3´	R
iglG_F	5´TTAAATATTATAAATGACTCCTTAAA3´	F
iglG_R	5´AGATGTTTTTACATTTATTTGTCC3´	R
iglH_F	5´GATGAAAAAAGAAAAGATTTAAGTA3´	F
iglH_R	5´TATAGAGTTATTTAAAACAATCTTTT3´	R
iglI_F	5´CCGCGGAAGTCAGATAATATCTACACTAAAT3´	F
iglI_R	5´CTCGAGTATGTCAAAAAGATCTTCAAAATA3´	R
iglJ_F	5´AAGACTATTTTGAAGATCTTTTTG3´	F
iglJ_R	5´TAAATTAAAATAACTTAGGTATATCT3´	R
pdpA_F	5´TTTGGACTAAGCACAAACCAT3´	F
pdpA_R	5´GTCATTATTAACATTTTCTCCAAT3´	R

pdpB_F	5´GCTATCTATAAAAGCTCTATAAA3´	F
pdpB_R	5´CAGTTTTATTATAAAAAAGTAGTG3´	R
pdpC_F	5´TATCTAAAGATATTATAAAATCATATA3´	F
pdpC_R	5´AAGCGTCAGCATATTTTTGTAA3´	R
pdpE_F	5´CCGCGGAAGTAAAAAAATATTTAAATTATTATCAA3´	F
pdpE_R	5´CTCGAGTATTATAGTAATTTTCTTTTCATAAT3´	R
vgrG_F	5´TCAAAAGCAGACCATATTTTCAA3´	F
vgrG_R	5´TCCAACCATTGTTGCTGTAGA3´	R
dotU_F	5´AAAGACTTTAAAGAGATAGAAATTA3´	F
dotU_R	5´CCAGCTTAATAAAATTAGTAAGC3´	R
pigR_F	5´ATGGCGAATCAATATTCTGGAA3´	F
pigR_R	5´CAGTCAAGATTTAGCTTTGATTA3´	R
rpoA_F	5´GTGAGTAATAATAATTCAAAACTG3´	F
rpoA_R	5´TTATTTTCCTTCAACTAGCTCTC3´	R
